# Review of the Foundational Knowledge Required for Assessing Horse Welfare

**DOI:** 10.3390/ani12233385

**Published:** 2022-12-01

**Authors:** Andrea M. Harvey, Daniel Ramp, David J. Mellor

**Affiliations:** 1 Centre for Compassionate Conservation, TD School, University of Technology Sydney, Ultimo, NSW 2007, Australia; 2 Animal Welfare Science and Bioethics Centre, School of Veterinary Science, Massey University, Palmerston North 4442, New Zealand

**Keywords:** horse, welfare assessment, wild horse, Five Domains

## Abstract

**Simple Summary:**

The key starting points for assessing horse welfare are a detailed understanding of what is usual for horses under optimal conditions, and knowledge about conditions that may impact welfare. This review presents the foundational knowledge required for undertaking comprehensive assessments of horse welfare organised according to a novel holistic framework, i.e., the Five Domains Model. It is a resource specifically tailored to assist those wishing to assess the welfare of horses in free-roaming or domestic situations.

**Abstract:**

A detailed understanding of what is usual for a species under optimal conditions is critical for identifying and interpreting different features of body function that have known impacts on animal welfare and its assessment. When applying the Five Domains Model to assess animal welfare, the key starting point is therefore to acquire extensive species-specific knowledge relevant to each of the four physical/functional Domains of the Model. These Domains, 1 to 4, address areas where objective information is evaluated and collated. They are: (1) Nutrition; (2) Physical environment; (3) Health; and (4) Behavioural interactions. It is on the basis of this detailed knowledge that cautious inferences can then be made about welfare-relevant mental experiences animals may have, aligned with Domain 5, Mental State. However, this review is focused entirely on the first four Domains in order to provide a novel holistic framework to collate the multidisciplinary knowledge of horses required for undertaking comprehensive welfare assessments. Thus, inferring the potential mental experiences aligned with Domain 5, the final step in model-based welfare assessments, is not considered here. Finally, providing extensive information on free-roaming horses enables a better understanding of the impacts of human interventions on the welfare of horses in both free-roaming and domestic situations.

## 1. Introduction

A detailed understanding of what is usual for a species under optimal conditions is critical to identifying and interpreting differences, and it is the body of knowledge that underpins the assessment of welfare. Furthermore, knowledge about conditions that may have positive or negative impacts on welfare, and how these may be recognised, is essential for interpreting welfare indicators. This welfare-focused information enables cautious inferences to be made about the animals’ mental experiences, which may be generated by its internal states and/or by its external circumstances, as captured by the Five Domains Model used for animal welfare assessment [1,2,3]. It is these cautiously inferred affective or mental experiences that are the ultimate focus of these welfare assessments, assigned to the Fifth Domain.

Such assessments of welfare require a holistic understanding of multidisciplinary knowledge. A Ten-Stage Protocol for applying the Five Domains Model for assessing welfare, using free-roaming wild horses as an example, has been published, and is summarized in Table 1 [4]. Stage 3 emphasises the importance of acquiring species-specific knowledge relevant to each of the first four Physical/Functional Domains of the Model, prior to determining which specific welfare indicators may be used reliably in such assessments. The Physical/Functional Domains are the areas in which objective information is evaluated and collated. They are: (1) Nutrition; (2) Physical environment; (3) Health; and (4) Behavioural interactions [3]. Harvey et al., 2020 highlighted what species-specific information was required to assess the welfare of free-roaming horses aligned with these four Domains (Table 2) [4]. This review has therefore been structured with the key points in Table 2 as headings. It is on the basis of this detailed knowledge that cautious inferences can then be made about welfare-relevant mental experiences animals may have, aligned with Domain 5, Mental State. However, Domain 5 is not included in this review and will be addressed directly in subsequent publications which are in preparation.

Consolidating key foundational knowledge of horses within each Physical/Functional Domain of the Model, provides the reference material required whenever assessment of equine welfare is being undertaken. The primary purpose of this review is to provide a comprehensive resource for those wishing to assess the welfare of horses in free-roaming or domestic situations. This review is unique in the collation of such comprehensive multidisciplinary knowledge from an animal welfare perspective. Importantly, the information on wild free-roaming horses is also required for a better understanding of the impacts of human interventions on the welfare of horses in both free-roaming and domestic situations. Finally, this review also provides guidance for researchers of other species by demonstrating the breadth and depth of information required in each case to undertake detailed welfare assessments.

## 2. Domain 1: Nutritionü

### 2.1. Water Requirements 

Daily maintenance water requirements for an adult non-lactating, non-pregnant horse at rest, and at thermoneutral temperature (see Domain 2) is approximately 50 mL/kg/day, i.e., for a 300 kg horse this would be 15 litres per day [5].

Domestic horses are observed to drink peri-prandially approximately 80% of the time, or 4 times daily when fed ad lib [6,7,8]. Normal drinking patterns for housed mature horses are episodic occurring 2–8 times per day [7]. Most wild horse populations are reported to drink at least once per day [9,10,11], with free-ranging Przewalski horses and some free-roaming horses in the US observed to drink twice daily [12,13]. In arid Australia, when distances are large between good forage and water sources, drinking as little as once every 48 h has been described [14,15,16]; some horses have been recorded to walk up to 55 km to watering points, with a mean drinking interval of 2.67 days (range 1–4 days) [15,16]. Free-roaming Przewalski horses drank less frequently post-release compared to in captivity when they drank about five times daily [17,18], suggesting that drinking frequency is affected by availability [19], and that water availability is not always optimal for free-roaming horses. 

#### 2.1.1. Factors Influencing Water Requirements 

The frequency of drinking and volume of water required varies with age, sex, weight, activity, reproductive status, health status, climate and diet [5]. Lactation is an important factor increasing water requirements. To offset lactational requirements, water intake needs to be increased by 30–60% above maintenance. Lactating mares in the wild have been observed to drink more frequently and therefore tend to remain close to water sources [20].

In wild horses, water requirements have also been shown to increase during dry conditions [11]. In domestic horses high ambient temperatures alone (>33 °C) increase evaporative fluid loss by 4–5 times in resting horses and by 45–60% during exercise [21,22,23]. Part of the acclimatisation to hot ambient temperatures involves changes in water intake and does not confer resistance to dehydration [24]. In one study water intake increased from approximately 23 litres per day at thermoneutrality (20 °C, 45–50% relative humidity) to about 40 litres per day after short term exposure to a hot humid environment (33–35 °C, 80–85% relative humidity) [25].

#### 2.1.2. Adaptations to and Impacts of Water Restriction

**Figure 1:**
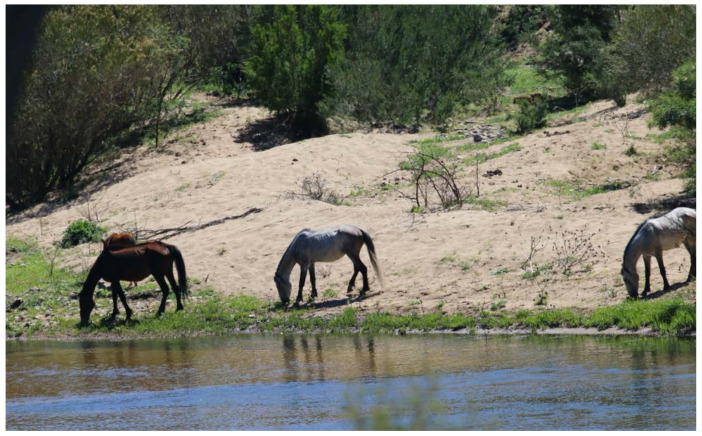
Wild horses grazing on the edge of the Lower Snowy River, NSW, Australia. Image A. M. Harvey.

Given horses’ dependence on water, especially in warm seasons, water distribution strongly influences the dispersal, space use and movement patterns of free-roaming horses [19]. When water sources are more abundant, horses spend less time moving (Figure 1). In regions where it snows during winter, horses can eat snow for hydration which potentially enables foraging further away from liquid water sources compared to summer months [26,27]. 

Water reserves in horses are more labile than energy reserves which means that horses tolerate water deprivation for much shorter periods than they can tolerate food deprivation. Water restricted horses spend less time eating with feeding activity gradually declining as water is restricted [28]. Reduced access to water is also a factor that has been shown to increase the risk of gastric ulceration in domestic horses [29]. However, no evidence of gastric ulceration was found in any wild horses studied in different populations in outback Australia [30], which is consistent with other risk factors also being important. 

Dehydration greater than 15% can be fatal for a horse [31]. On average this would occur after approximately 7 days of water deprivation depending on environmental conditions [5].

### 2.2. Nutritional Requirements and Preferences 

Horses are predominantly grazers and typically select graminoids over other species, with one study finding over 35 different grass species in the diet of free-roaming horses [14]. Forbs and shrubs are less frequently consumed, more commonly in winter, and usually only comprising a small proportion of the diet [32,33,34]. A potential reason for this is the high concentration of phenolics in forbs and shrubs, as equids do not have the capacity to detoxify plant phenolic compounds [35,36].

**Figure 2:**
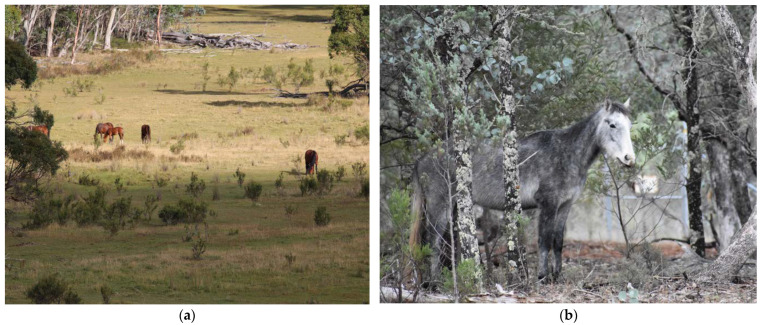
Horses grazing in different habitats in Kosciuszko National Park, Australia: (**a**) Horses are predominantly grazers; however, (**b**) shrubs and trees form a variable proportion of their diet, increasing when availability of grass is limited. Images A. M. Harvey.

Several studies report grasses making up to at least 83% of the diet of free-roaming horses in all seasons [37,38,39], although in other populations only 56% of the diet comprised grass species with the remainder of the diet consisting of sedges, rushes and browse [37,40] (Figure 2). Some authors report consistent diets in free-roaming horses throughout the year whilst others report seasonal differences due to changes in forage availability [34,37,38,39,41]. Horses in the Australian desert were found to have up to 28% of their diet as dicotyledonous species and observations of horses eating the tops of shrubs and trees were common when grass growth ceased [14]. 

Grazing studies have suggested that horses will select different qualities of forage when given opportunity, and the extent of the grazing resource and its diversity will affect the qualitative intake [42,43,44]. When horses were offered a choice test, they appeared to select a higher energy diet [45]. Food flavour and odour have also been noted to influence food choice in horses [46]. Free-ranging Konik horses grazing natural vegetation selected about 50% of their diet based on the intake of digestible organic matter, a measure that is the product of bite size, bite rate, and bite digestibility from plants with the highest content of digestible organic matter [47]. So, when given the opportunity horses will select dietary components that will maximise their energy intake over time. Further studies have suggested that diet selection can be influenced by grass quality as horses remained grazing short patches of quality pasture rather than utilising longer swards of inferior grasses [42]; others also report preferences for short grasses of lower bulk density rather than taller grass on perennial ryegrass swards [43]. It is also reported that fibre content of the diet influences colonic dry matter, volatile fatty acid production and particle size [48]. Data collected from stomach contents at post-mortem suggested that food resource quality cannot be determined only by the physical characteristics of the apparent forage [30]. 

### 2.3. Common Nutritional Deficiencies and Excesses and Their Causes 

Lack of rainfall and overgrazing can result in significant dietary protein deficiency [30], which when prolonged may result in weight loss, abortion and reduced milk production in pregnant and lactating mares [49]. Starvation, when health is impaired because of inadequate nutrition [50], is discussed in more detail under Domain 3. 

**Figure 3:**
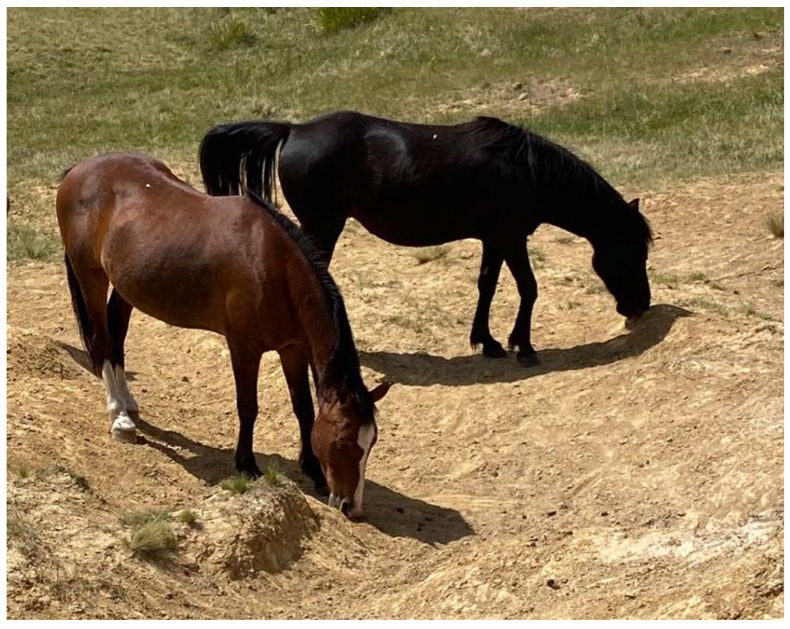
Wild horses exhibiting geophagia. Image A. M. Harvey.

In a post-mortem study of free-roaming horses in semi-arid Australia, dietary copper deficiency was present in all horses sampled at different sites, although no associated pathology was observed [30]. Copper is essential for normal collagen biosynthesis and skeletal development [51,52]. Foals born to mares with copper deficient diets have increased incidence of developmental orthopaedic disease [51,53], and in adult horses copper deficiency has been linked to osteoporosis, joint effusion and limb deformity, and secondary iron deficiency anaemia [51]. Both iron and copper deficiency have also been identified as potential causes of geophagia (Figure 3) and pica in horses [54,55]. 

Secondary hyperparathyroidism (‘big head disease’) is recognised in horses arising from excessive phosphorus intake coupled with inadequate calcium intake, resulting in calcium mobilisation from bone [56], and can be associated with plant calcium oxalate toxicosis (see Section 2.4 below). In the semi-arid Australia post-mortem study, evidence of wide variation in dietary phosphorus, magnesium and sodium, was found, with some levels being below recommended daily intakes [30]. With phosphate deficient diets, phosphorus is mobilised from bone to maintain blood and tissues concentrations [56]. Inadequate magnesium intake can be associated with poor growth, increased disease risk due to immune-incompetence, and muscle weakness [56]. Most feeds of plant origin are low in sodium and inadequate sodium intake in horses is common, with signs being increased licking and searching for salty substances [56].

Dietary zinc concentrations were also very low in the Australian study [30] and low soil zinc concentrations are known to be prevalent in some regions [56]. Zinc deficiency has been linked to developmental orthopaedic disease and reduced hoof horn strength and hardness [56,57]. High dietary ash content was also found, suggesting that the horses may have consumed soil [30], a common occurrence in horses grazing sandy soils [58] and at natural mineral licks [37]. Ingestion of soil or soil contaminated vegetation may also increase iron intake and may explain both the high dietary iron and cobalt that was identified [30,56].

### 2.4. Plant Toxicities

Plant toxicities most commonly occur in conjunction with undernutrition, since horses will most likely only ingest toxic plants when there is an inadequate alternative source of food. Diagnosis usually involves a post-mortem, and even then, some are difficult to definitively diagnose. Presumptive diagnosis may be made when a horse presents with consistent clinical signs in an environment with a high density of the toxic plant. There is a very wide range of plants that can cause toxicity in horses, and this can vary geographically and between different environments and habitats. Familiarisation with common and unique plants in the environments in question is therefore crucial. 

**Figure 4:**
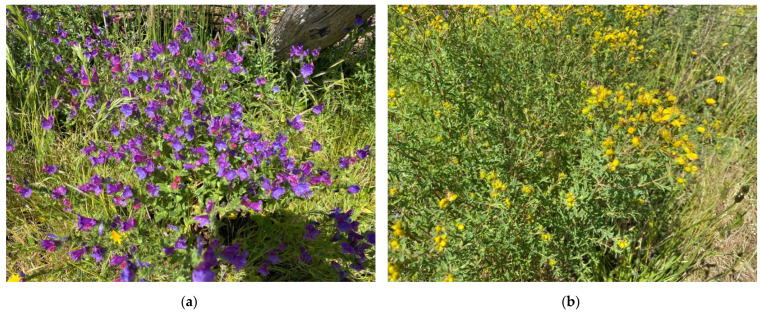
Examples of toxic plants (**a**) Patterson’s curse (*Echium plantagineum*) is a cause of pyrrolizidine alkalosis; (**b**) St John’s Wort (*Hypericum perforatum)* is a cause of photosensitisation. Images A. M. Harvey.

The most common toxicities are related to pyrrolizidine alkalosis [e.g., Patterson’s curse (*Echium plantagineum*) and Fireweed (*Senecio madagascariensis*)] (Figure 4a), photosensitisation [e.g., St John’s Wort (*Hypericum perforatum*)] (Figure 4b), calcium oxalate toxicosis [e.g., Purslane (*Portulaca oleracea*), Lambsquarter (*Chenopodium album*), Kikuyu grass (*Pennisetum clandestinum*), Setaria grass (*Setaroa sphacelate*)], selenium toxicosis [accumulated in a range of plants (e.g., *Astragalus*, *Stanleya*, *Machaeranthera*, *Oonopsis*, and *Xylorhiza*) when growth is in alkaline soils with low rainfall], and thiaminase toxicosis [e.g., Bracken fern (*Pteridium esculentum*)]. Pyrrolizidine alkalosis causes cumulative toxicity eventually resulting in chronic liver failure with diarrhoea, weight loss, subcutaneous oedema, neurological signs and ultimately death. Photosensitisation results in skin lesions, typically on sensitive areas or unpigmented skin. Selenium toxicosis results in hair loss and severe hoof pathology leading to lameness. Thiaminase toxicosis results in neurological signs and ultimately death. Calcium oxalate toxicosis results in nutritional secondary hyperparathyroidism with a relative calcium deficiency and subsequent fibrous osteodystrophy and osteoporosis, frequently presenting as ‘big head’ [59]. Plant toxicity is also therefore relevant to Domain 3 in terms of associated health problems and potential sickness behaviours.

Some disorders secondary to plant toxicities specifically reported in Australia include ‘Australian Stringhalt’, a peripheral nerve disorder that has been associated with ingestion of flatweed (*Hypochaeris radicata)* [60], and neurological disease associated with ingestion of *Indigofera enneaphylla* in subtropical and arid regions of Australia [61]. The study of free-roaming horses in central Australia also identified high concentrations of cobalt in the stomach of horses in five different environments, with one hypothesis being consumption of native plants that accumulate cobalt [30]. Identified in central Australia, is a small shrub violet (*Hybanthus floribundus*) that accumulates high concentrations of nickel and cobalt [62], and different species of this shrub have also been identified more widely in Australia [63]. The requirements and toxicity of cobalt have not been studied in horses [56], but these findings illustrate the importance of having local knowledge of vegetation and an awareness of knowledge gaps. 

### 2.5. Body Condition Scoring 

Body condition scoring (BCS) is a standardized semi-quantitative method to visually evaluate the amount of fat and muscle in a horse’s body; it takes into account the deposition of body fat in different areas by examination of the neck, back, ribs, pelvis and rump [64]. Scoring systems have been well described using either a 9-point scale, where 1 is emaciated and 9 is extremely fat [65], as illustrated in Table 3, or a 6-point scale where 0 is emaciated and 5 is very fat [64]. Body condition scoring has been widely used in horses [64,65,66,67,68,69,70,71,72,73,74,75] and, in experienced hands, is a consistent and reliable method of estimating body condition [76,77,78,79]. 

#### 2.5.1. Optimal Body Condition Score

On the 9-point scale, optimal body condition is 5–6, with BCS ≤3 being underweight [80]. Mares with body condition of less than 5 have lower reproductive performance, with delayed onset of oestrous and ovulation, reduced conception rates, higher numbers of cycles to conception, and reduced maintenance of pregnancies [66]. Mares of BCS 3–3.5 also had extended seasonal anoestrous compared to mares of BCS 7.5–8.5 [81]. Similarly, it has also been suggested that stallions have the best reproductive success at body condition scores of 5–6 [82].

In addition to influencing conception and birth success, body condition and resource availability may also influence the sex of offspring, with mares in poor condition at the time of conception producing more female foals [83]. In a resource limited population of wild horses in another study, a ratio of 0.47 male foals to 1 female was observed [84]. 

Maternal body condition has also been shown to influence the amount of time mothers with foals at-foot spent vigilant, with mares in good condition spending more time vigilant than mares in poor condition [85]. Mares in good condition can afford to spend more time alert at the expense of feeding time, whereas mares in poor condition must spend more time foraging [86], impacting on ability to invest in the protection of their offspring. 

Impacts of low body condition scores on health and reproduction are discussed in Domains 3 and 4. 

#### 2.5.2. Factors Affecting Body Condition 

Increased energy demands will negatively impact body condition if they are not met by adequate increases in food intake. Common causes of increased energy demand include late pregnancy and lactation, with mares commonly having lower BCS than stallions [30,70,87], increased activity which may occur with stallions during the breeding season, horses that must travel long distances between food and water sources [30], and cold weather [24]. Disease may also interfere with digestion or increase nutrient utilization, such as in the case of endoparasitism, which has been associated with low body condition scores in one wild horse study [88], but not in another study [89].

Reduced food intake may occur with reduced food availability, related to low rainfall or populations reaching carrying capacity [30,90]. In individual horses, injury or disease may also reduce foraging activity. 

Forage quality may also impact body condition. In a study in semi-arid Australia all horses were judged to be moderately thin (mean BCS 3.9 out of 9) across five populations [30]. The dietary protein concentration in stomach contents of these horses varied from 4.3% to 14.9% and was found to be significantly correlated with body condition score, with thinner horses having lower protein concentrations in stomach contents. Conversely, concentrations of water soluble and ethanol soluble carbohydrates in horse stomach contents were well above those expected even for high quality forage. When combined, these observations suggest that the diet of these horses was suboptimal [30]. 

Foraging behaviour is also relevant to this Domain and is discussed under Domain 4. Habitat and weather are intrinsically related to forage availability, as discussed under Domain 2. Health problems may affect body condition, and conversely mal/under-nutrition can cause health problems, and these are discussed under Domain 3. 

## 3. Domain 2: Physical Environment

### 3.1. Habitat Preference and Factors Affecting Habitat Use

Habitat selection and use are primarily influenced by forage availability and quality [91,92], with distance to water also being important [93]. The primary determinant of habitat use in free-roaming horses in all seasons has been shown to be availability of preferred forage [34,37,94,95,96]. Intermittent drought and changes in rainfall can create a great deal of seasonal variation in quantity and quality of available forage which may impact habitat use. 

In New Zealand changes in habitat preference coincide with the beginning of foaling and mating in spring and formation of frost inversion layers in winter [97]; in addition, free-roaming horses occupy lower altitudes on gentler slopes, river basins and stream valley floors in spring and summer, compared to steeper slopes at higher altitudes in winter [97]. Despite the ability of horses to obtain forage beneath the snow, the presence of snow limits habitat use [37]. Horses in the Australian Alps are limited in distribution in winter, avoiding deep snow at higher elevations [98]. 

Home range is highly dependent on resource availability and varies considerably within and among populations [99], with reported home range sizes varying from 6–303 km^2^ [95,96,100,101]. Bands generally have high fidelity to home ranges [92]. Band sizes have been correlated to home range size indicating that it is likely related to resource demand [97]. 

Horses travel between feeding and resting spots to seek dry ground to lie down or resting places that provide refuge relief from insects [102,103,104,105,106]. They also travel between feed and water sources, sometimes for long distances [15]. In one study in Central Australia and Queensland, the average distance travelled was 15.9 ± 1.9 km/day (range 8.1–28.3 km/day) [15]. Central Australian horses watered less frequently and walked for long distances in direct lines to patchy food sources, whereas central Queensland horses were able to graze close to water sources and moved in a more-or-less circular pattern around the central water source [15].

### 3.2. Preferred Underfoot Substrate and Terrain

As described above, habitat selection appears to be primarily driven by resource availability rather than underfoot substrate and terrain. Free-roaming horses reside in both flat and steep mountainous terrains, and a range of surfaces from rocky to wetlands. The effects of the environment on the feet of free-roaming horses have been well studied in Australia [107], with the hardness of underfoot substrate and travel distance affecting foot morphology [108] (discussed more in Domain 3). 

### 3.3. Thermoneutral Zone

Wild horses exist across many different environments, and are very adaptable to temperature variation, thriving in climates ranging from tropical to subarctic [109]. In Australia alone their habitats vary from semi-arid Central Australia to the snow-covered mountains of the Australian Alps [14,30,85,110]. The potential environmental challenges encountered by horses will thus vary greatly between these different habitats. 

The thermoneutral zone (TNZ) is defined as the range of effective ambient temperature where internal thermostability is maintained without changes in metabolic heat production [24,109]. It comprises three zones: cool, optimal and warm [24]. The lower end of the TNZ is known as the lower critical temperature (LCT) and is the ambient temperature below which metabolic heat production increases, so that without extra feed, feed energy is diverted to heat production rather than tissue growth or maintenance. The upper end of the TNZ is known as the upper critical temperature (UCT) at which evaporative heat loss increases via sweating to prevent core body temperature from rising [24]. 

**Figure 5:**
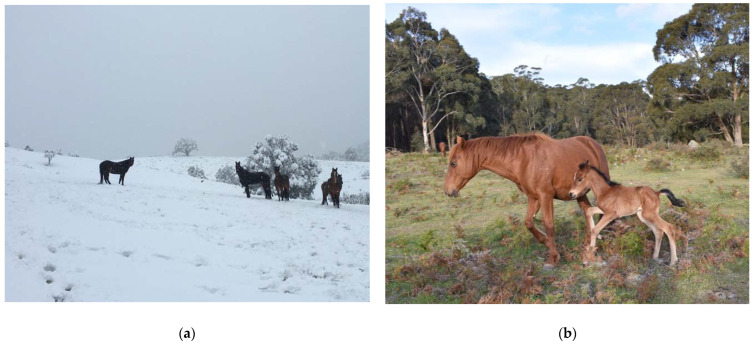
The thermoneutral zone of horses can vary. (**a**) Horses can adapt to different climates and modify their thermoneutral zone. (**b**) Newborn foals have much higher lower critical temperatures [24,109]. Images A. M. Harvey.

The thermoneutral zone for horses has been defined as 5–25 °C [111]; however, horses can adapt to different climates and modify their thermoneutral zone [24]. For example, acclimatised adult horses have been documented to have a LCT as low as −15 °C [109]. The LCT also varies with seasons, breed, age, body condition and intake of dietary digestible energy [109]. For example, the LCT for newborn foals is much higher, reported to be around 14–22 °C, reducing gradually during the first week after birth [24,109] (Figure 5).

### 3.4. Thermoregulation and Factors Affecting It 

Thermoregulation in cold weather is achieved through increases in coat insulation, as well as changes in posture and other behaviours, heart, respiratory and metabolic rates, and thermoregulatory hormone secretion [109]. Shelter seeking and huddling occur with acute cold. Foraging and movement also decreases and may conserve 17% of daily energy expenditure [24]. 

Other factors such as body condition, food intake, having a wet coat, access to shade/shelter and access to water will also influence how well the animal may tolerate thermal extremes. Microclimatic variables are ambient temperature, relative humidity, precipitation, wind velocity and solar radiation, with ambient temperature being the most important [24]. Being wet increases evaporative heat loss, directly reduces thermal insulation of the hair coat and increases conductive heat losses to the cold rainwater [24]. Snowfall is less cooling than rain because, in the absence of wetting, the hair coat retains a significant capacity to insulate against heat loss [24].

At LCTs horses rely on insulation and food intake to maintain body temperature [24]. Insulation is derived from muscle, fat, skin and hair with subcutaneous fat being particularly important. Thus, thermoregulatory ability is negatively impacted if horses are in poor body condition and/or if food availability is restricted [24]. For example, acclimatised yearlings fed ad lib had a LCT of −11 °C but those fed for normal growth (with limited food access), had a higher LCT of 0 °C [109].

As temperature approaches UCT, when available, horses will seek shade where radiant heat loads are lower, limit their movement to reduce internal heat production, and may enter water holes or rivers where immersion in water has direct conductive cooling effects and increases evaporative heat loss when they exit the water. The circulatory system and sweat glands act as major thermoregulatory mechanisms. Peripheral vasodilation enhances blood flow to the skin increasing convective heat loss and sweating leads to evaporative heat loss [112]. Sweating rate will continue proportionate to rises in core body temperature, but at very high ambient temperatures evaporation may not keep pace with the rate of sweating, making evaporative heat loss less effective, and also leading to large fluid losses. However, this is usually only an issue during protracted moderate-to-high intensity exercise [112].

### 3.5. Impacts of Ambient Cold 

Cold and/or wet weather increases energy requirements and can be associated with weight loss. Metabolic rates increase by 70% above resting levels with severe cold exposure [109], which results in weight loss, or reduced weight gain in growing animals, if energy intakes are restricted. Young horses kept at −5 °C had 29% lower average daily weight gain than those kept at 10° C [24]. Therefore, energy intake needs to be increased during cold exposure to avoid weight loss in mature horses, and reductions in weight gain in growing young. At temperatures below LCT, maintenance energy intakes should be increased by 2.5% per °C decrease in effective ambient temperature for adult horses, and by 1.3% for growing horses [109]. 

Horses in poor body condition need significant additional feed to gain weight in colder temperatures. For example, at −10 °C, a 500 kg horse needs 16.4 MCal/day digestible energy (equates to approx. 9 kg of hay) to maintain body weight, whereas a thin horse with a body condition score <4 (nine-point scale) needs 26.4 MCal/day digestible energy (14kg + hay) to gain 0.5 kg in body weight [24]. Acute cold exposure also alters some serum biochemical parameters reflective of an enhanced mobilisation of fat and protein to fuel cold-induced increases in thermogenesis [24].

### 3.6. Impacts of Ambient Heat 

With protracted intense exercise during hot and/or humid conditions, thermoregulatory failure may occur. Once core body temperature reaches 42.5 °C without effective cooling, hyperthermia-induced pathological changes develop [112]. Severe fluid and electrolyte losses occur, and horses become fatigued, tachypnoeic and tachycardic. Fluid and sodium loss associated with excessive sweating results in hypotonic dehydration and affected horses can lose their desire to drink. Potentially fatal metabolic disturbances can follow, including exertional rhabdomyolysis, synchronous diaphragmatic flutter, gastrointestinal stasis, colic and renal failure. This can progress to ataxia, collapse, convulsions, coma and death [112]. 

### 3.7. Impacts of Extreme Climatic Events 

The effects of catastrophic environmental factors on mortality are described under ‘population dynamics’ in Domain 4, but climatic events such as drought, storms, heavy snow, extreme cold, thunderstorms, lightning strikes and fire have all been reported to cause mass mortalities in wild horse populations [14,113,114,115,116]. 

## 4. Domain 3: Health

It is beyond the purposes of this review to discuss health problems of horses in any depth, so the aims here are to: (i) briefly summarise the predominant health problems reported in the literature; (ii) emphasise those that are likely to be common in wild free-roaming horses; (iii) highlight areas where there may be significant knowledge gaps; and (iv) indicate important sources of further information. 

The most common health problems in wild horses may be anticipated to vary geographically between different countries and different environments, as well as between different age groups. Anyone involved in assessing equine welfare should familiarise themselves with clinical signs, risk factors, aetiologies, and the diagnosis of and prognosis for key diseases mentioned below, and especially for those specific to their region.

### 4.1. Common Non-Infectious Diseases 

Aside from injuries, to which US wild horses are said to be susceptible, the most common non-infectious health problems are those secondary to limitations in food and water, followed by age related disease, club feet, and developmental orthopaedic disease with angular and flexural deformities being most common, albeit rare overall [117,118,119]. Age related development of osteoarthritis in the metacarpophalangeal joints of wild horses in New Zealand has also been identified [120]. 

**Figure 6:**
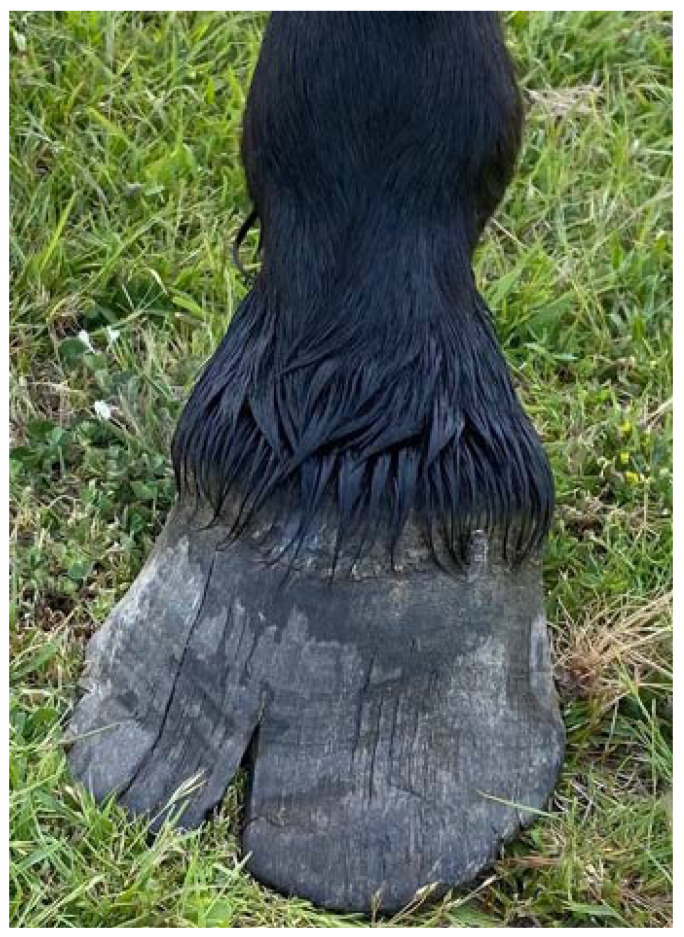
Hoof pathology such as cracks may be common in some populations, but relationship to lameness is not clear. Image A. M. Harvey.

In free-roaming horses in a Polish sanctuary, the most common health problems noted over a 70-year period of monitoring, were hoof pathology (Figure 6) and parasitic diseases [121]. However, resultant lameness, or clinical signs associated with parasitism were rare. Undernutrition was also rare since the small population was maintained at a stable number and supplementary feeding was carried out if needed [121]. Reported causes of mortality are described under Population Dynamics in Domain 4.

However, evaluation of the peer reviewed literature suggests that health problems are a key knowledge gap in wild horses. Common health problems likely to be encountered in wild horses are difficult to extrapolate from the extensive knowledge of health and disease in domestic horses, since domestic horse health is most often related to husbandry, particularly diet and environmental factors. Poor forage availability can predispose to conditions such as colic, gastric ulceration, and abnormal dental wear, whilst over-nutrition is common resulting in obesity, equine metabolic syndrome and related disorders such as laminitis. Restrictions on movement can have negative impacts on hoof health and musculoskeletal development in addition to predisposing horses to obesity and related diseases. An indoor dusty environment is a major contributor to lower airway disease. The most common health problems in older domestic horses are cardiovascular disorders, locomotor system injuries, hoof abnormalities, dental disease, weight loss and colic [122,123]. 

Conversely, the only health related area that has been extensively researched in wild horses is foot pathology in Australian desert horses [107,108,124,125], and free-roaming horses in New Zealand [126,127]. The most striking finding in these studies was a high prevalence of chronic laminitis in these populations. This may be surprising since laminitis is more typically associated with diet and husbandry issues in domestic horses [128]. This pathological finding in wild horses could suggest diets high in non-structural carbohydrates, or presence of insulin resistance [127]. However, this pathology in horses walking long distances on hard surfaces is likely a result of foals having to travel long distances early in life, impacting the early development of the hoof [107,129]. As these studies were conducted at post-mortem, the clinical relevance for the prevalence of lameness associated with these findings, is unknown. In contrast, Sable Island horses that reside on a soft sandy substrate have a low prevalence of laminitis but overgrown hooves are common [130].

Food limitation may give rise to other health problems. Specific nutritional deficiencies and related disorders were discussed under Domain 1. When normal food sources are limited, horses are more likely to ingest toxic plants, also discussed under Domain 1. Soil ingestion may be more likely to occur in environments where forage is sparse [30], or with some nutritional deficiencies (See Domain 1). In some circumstances this may give rise to sand enteropathy [131], although this has not been specifically documented in wild horses. Low body condition is also associated with decreased reproductive performance, with widespread effects on reproductive physiology of mares [132].

There is limited information in horses indicating their susceptibility to different diseases when they are malnourished, but it is well established in humans that malnutrition increases risk of disease and decreases survival rate [133]. Acute starvation occurs when food intake ceases or is exceptionally low, leading to a rapid onset of emaciation, and related impairments to health [50]. In horses, systemic immunity may be severely compromised within 5 days of the onset of starvation [134]. Chronic starvation gives rise to a range of metabolic changes that reduce basal energy requirements, with eventual sequalae of reduced body mass, reduced lower critical temperature, reduced integrity of the gut mucosa, gastric ulceration, and compromised immunity resulting in increased risk of infectious diseases, and endoparasitism [135]. Chronic starvation is associated with decreased levels of activity to only essential movements in people [136], and similar effects may be expected in horses, with death typically occurring after a body mass loss of about 50% [5]. Death by starvation has been reported in large numbers of wild horses in semi-arid habitats during droughts [14,137].

**Figure 7:**
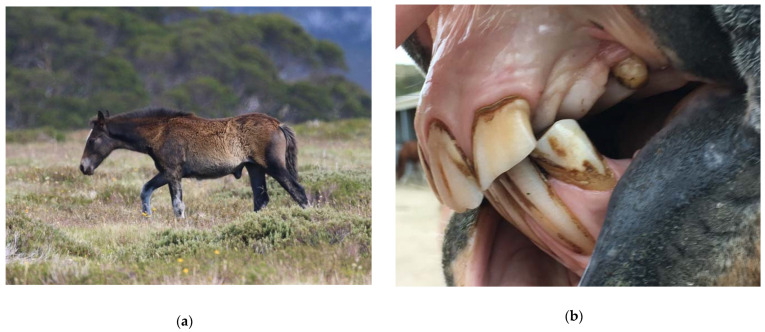
Examples of pathologies that may be genetic in origin. (**a**) Ventral abdominal hernia in a young wild horse in the Victorian Alps, Australia. (**b**) ‘Parrot mouth’ in a horse removed from Guy Fawkes National Park, NSW, Australia. Images A. M. Harvey.

Sufficient genetic diversity is important for the prevention of inbreeding and subsequent development of genetic diseases. The population size necessary to prevent genetic problems from arising in horses is not known, but it will depend on the genetic diversity of the population. Crude estimates of 50 breeding horses or a total of 150 horses in a population have been suggested to minimise the risk of genetic disorders [138]. Genetic disorders including club foot deformity, dwarfism or blindness associated with cataract formation, ventral abdominal hernias and cryptorchidism have occurred in the US [117]. One of the authors (AH) has also observed ventral abdominal hernias, ‘parrot mouth’ (Figure 7), pelvic limb deformities and cryptorchidism in Australian wild horses, which may be genetic in origin (personal observations). 

### 4.2. Common Infectious Diseases

**Figure 8:**
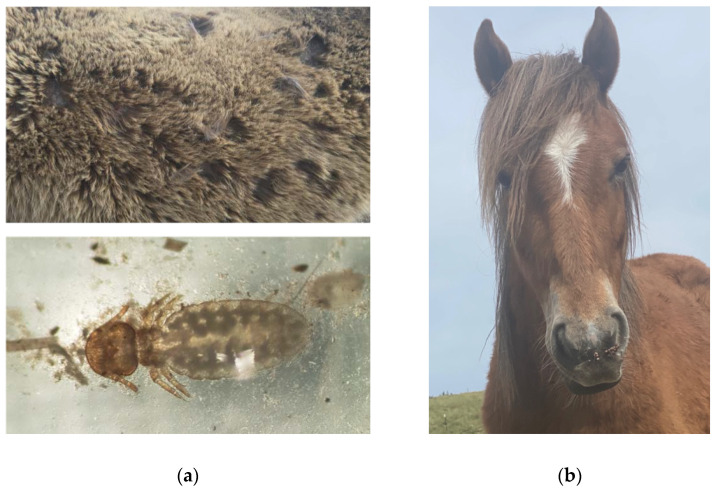
Examples of skin lesions caused by infectious diseases. (**a**) Skin lesions (upper image) caused by the louse *Damalinia equi* (lower image). (**b**) Warts on the nose caused by Papillomavirus. Images A. M. Harvey.

Specific infectious disease risks will vary considerably between different geographical regions. In Australia, the main infectious diseases of potential concern would be gastrointestinal parasites, external parasites such pediculosis (lice) caused by *Damalinia equi* (Figure 8a) and *Haematipinus asini* [139], bacterial infections such as *Clostridium tetani* [140], *Streptococcus zooepidemicus* and *S. equi* [141], and viral infections such as Papillomavirus (Figure 8b) [142], Equine Herpes Virus [143], Ross River Virus [144], Hendra virus [145] and Lyssavirus [146]. However, no investigation of these infectious diseases has been performed in Australian wild horse populations. Although they will not be discussed here, readers can find more information in the texts cited above. 

Infectious upper respiratory tract diseases caused by *S. zooepidemicus* and *S. equi* have been documented in free-roaming wild horses in the US, however they appear to rarely cause a problem in free-roaming horses. They are mainly an issue when large numbers of horses are brought into a smaller area, or when many of them congregate spontaneously in one area, for example during times of water or feed shortage [117,119]. In the US, clinical problems have been observed in wild horses with Eastern equine encephalitis virus, West Nile Virus, and equine infectious anaemia, but this appears to be rare [117]. Equine protozoal myeloencephalitis caused by *Sarcocystis neurona* occurs in some countries including the US, and seroprevalence has been determined in wild horses in Wyoming [147], although the incidence of associated clinical disease has not been reported. In Venezuelan wild horses, muscular disease associated with *Trypanosoma evansi* has been identified [148]. Otherwise, very little is known about the prevalence or significance of infectious diseases in wild horse populations throughout the world.

Gastrointestinal parasitic diseases are the most studied to date, but the reports have been predominantly anecdotal descriptions of high parasite burdens at post-mortem [30], and in horses removed from the ranges in the US [118]. Nevertheless, there are a few studies of gastrointestinal parasites in wild horses worldwide [88,89,149,150,151,152,153]. Recently a high prevalence of the most pathogenic large strongyle, *S. vulgaris*, was identified across several wild horse populations in Australia [152]. 

**Figure 9:**
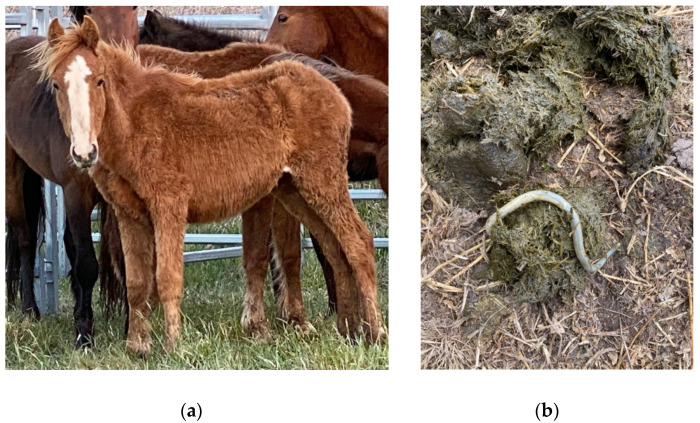
*Parascaris* spp. most commonly cause pathology in young horses (**a**) Approximately 6-month-old filly removed from Kosciuszko National Park, NSW, with poor body condition and a rough hair coat. (**b**) Following administration of anthelmintics she passed several large *Parascaris* spp. Images A. M. Harvey.

Gastrointestinal parasites described are most commonly ascarids, and small and large strongyles. *Parascaris* spp. most commonly cause pathology in horses aged < 18 months due to development of very strong acquired immunity in older horses, although anecdotally significant burdens have also been reported in the US in adult horses removed from the range [118,119]. Migration through pulmonary tissues can result in coughing and a purulent nasal discharge, whilst parasites in the gastrointestinal tract can result in reduced food intake, diarrhea, poor growth, poor weight gain, and a rough haircoat (Figure 9) [154,155].

Larval cyathostominosis, when large numbers of small strongyle (cyathostomins) larvae emerge from cysts in the intestinal wall, is associated with severe haemorrhagic oedema and inflammation in the large intestine [156,157,158], often associated with profuse diarrhea, weight loss, anaemia, hypoproteinaemia and accompanying ventral oedema and dehydration. This is most common in young horses, with reported mortalities of up to 50% in domestic horses [156]. Chronic cyathostominosis can be related to the presence of various stages of parasite’s life cycle and can cause weight loss, dull haircoat, pot-bellied appearance, colic, and mild diarrhoea [156]. The most pathogenic large strongyle is *S. vulgaris*, a cause of verminous arteritis in the cranial mesenteric artery. *S. vulgaris* was reported to be the cause of death in 10–33% of domestic horses presenting with abdominal crises, prior to the availability of macrocyclic lactone anthelmintics [159,160]. Other clinical signs associated with adult strongyles include caecal ulcers associated with anaemia, weight loss, and poor coat condition. Acute signs following infection with large numbers of larvae can include pyrexia, inappetence, malaise, weight loss, constipation or diarrhea, and can progress to abdominal pain, recumbency and death, commonly fatal in young foals [160]. Chronic infection from repeated smaller doses of larvae, may result in pyrexia, poor weight gain and intermittent episodes of abdominal pain [160]. 

### 4.3. Common Injuries

Injuries are common in domestic horses. These are often sport related injuries, paddock injuries associated with fencing, excessive activity after periods of confinement, closer confinement with other horses that may not be in stable social groups, and kick injuries, made worse if a kick is from a shod horse [161]. Injuries may therefore be less common in wild horses, however the incidence of injuries in free-roaming wild horses under different conditions is unknown. Steep uneven terrain may be more likely to increase the risk of ligament and tendon injuries. Lower limb fractures [117] and poorly healed old injuries [119] are most observed in US wild horses.

### 4.4. Sickness and Pain Behaviours 

Sickness and pain behaviours can be quite subtle, so detection relies upon a detailed understanding of usual behaviours. Sickness behaviours can be any change in usual behaviour, emphasising the importance of species-specific knowledge under optimal circumstances. Subtle changes in time budget behaviours may indicate presence of pain with reduced mobility, grazing, alertness and social interactions and have generally been associated with welfare compromise in domestic horses [162,163,164]. Although behaviour and time budgets have been well studied in wild horses (reviewed in [165]), time budgets under different conditions in the wild have not.

Wild horses usually regularly scan the environment and remain vigilant, responsive to environmental stimuli and ready to react to unusual stimuli [166]. Sick horses are often described as apathetic or depressed [67,69,167,168], characterised by prolonged periods of reduced mobility, head-lower-than-withers body posture, reduced responsiveness to environmental stimuli and self-isolation. A fixed stare, dilated nostrils and clenched jaw have also been associated with apathy [162]. In addition, a dull demeanour, reduced alertness often accompanied by self-isolation and lowered head carriage have been described with non-specific and abdominal pain [169,170], features often observed in combination with a ‘pain face’ grimace [171,172]. 

Furthermore, some behaviours that could be regarded as sickness behaviours may not be widely recognised as such. For example, coprophagia (ingestion of faeces) has been described as a usual behaviour in wild horses [19], but it has not been well described in domestic horses that are adequately fed. Interestingly, coprophagia seen in captive plains zebra and Przewalski horses housed in a grassless paddock with no other vegetation, resolved when vegetation was provided, and when they were housed indoors with ad lib hay [173]. This illustrates how some behaviours or clinical findings that may appear to be ‘natural’ are not necessarily signs of optimal welfare. In this situation, it is apparent that the knowledge gained from domestic horses and wild equids in captivity provides the helpful insight that coprophagia may be suggestive of inadequate provision of food. Interestingly, it has also been found that horses that developed equine motor neuron disease were more likely to be coprophagic than unaffected horses [174]. This association is due to both conditions being related to limited grass intake, the link being that equine motor neuron disease is associated with vitamin E deficiency and that a major source of vitamin E is fresh forage [175]. This condition usefully illustrates how features in all four physical Domains can interact: Domain 1, inadequate nutrition linked with Domain 2, environmental impairment of forage growth, Domain 3, health and vitamin E deficiency causing equine motor neuron disease, and Domain 4, the sickness behaviour of coprophagia being exhibited. 

## 5. Domain 4: Behavioural Interactions

### 5.1. Social Organisation and Factors Affecting It

#### 5.1.1. Social Structure

**Figure 10:**
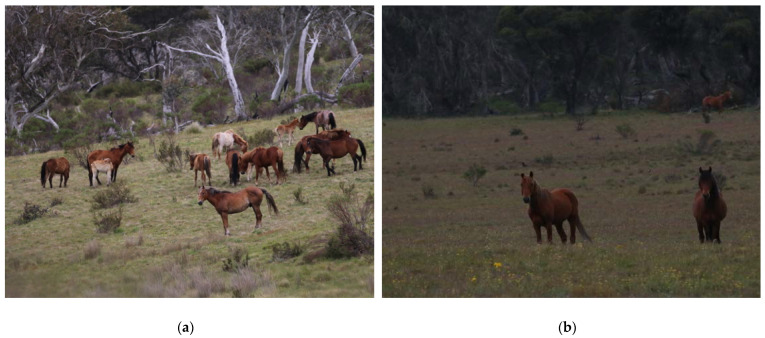
Horses are highly social animals exhibiting a harem social structure: (**a**) the social unit, known as a band, and (**b**) a group of bachelor stallions, in Kosciuszko National Park, NSW, Australia. Images A. M. Harvey.

Horses are highly social animals exhibiting a harem social structure and usually, long-term stability of social groups [97,176,177,178]. The social unit, known as a band, usually consists of one or more stallions, one or more mares, 1–2-year-old horses of both sexes that have not yet dispersed, and foals [97] (Figure 10a). Mean reported band size is 4–12.3 horses, with maximum band sizes of 8–24, whilst number of adult mares in a band has been reported to be a mean of 2–4 and maximum of 8 [87,95,97,179,180]. Wild horse populations with a female biased sex ratio have been described to live in larger groups with a mean band size of 14, maximum of 18–35, with a mean number of adult mares being 5.6 and maximum 8–22 [179]. The largest reported band sizes have been in populations with strongly female-biased adult sex ratios, with 28 on Assateague Island, USA and 35 in Hato El Frio, Venezuela [179,181]. 

Stallions not in a band, are usually young or displaced older stallions that form all-male bachelor groups [176,179,180] (Figure 10b). All-female and mixed-sex groups have been described in free-roaming horses on Assateague Island, USA [179], and in Kaimanawa mountains, New Zealand [97], but these are uncommon and usually temporary groupings. Solitary horses would typically be band stallions who have lost their females, and they are often old or sick [176,182,183]. 

An alternative social structure is observed in some other equid species, where the social group is dynamic with no long-lasting bonds other than between mares and their current foal [93,176,177,178,182]. In this social structure stallions are territorial and sometimes alone, or at other times they have temporary associations with mares; although mares are rarely alone, their social associations are only temporary. Wild horses do not typically exhibit this social structure, but a rare scenario has been described in free-roaming horses in Shackleford Banks Island, USA where different social systems were present in the same population, under different environmental conditions. Here, a female-biased sex ratio reduced the number of potential rivals whilst an open habitat made detection of other stallions easy, so the stallion could defend both mares and his territory without being with a band all the time. When the vegetation later changed to more uniform cover, social structures of all horses reverted to harems with overlapping home ranges [178,182]. 

#### 5.1.2. Factors Influencing Band and Herd Size

The size of bands is influenced by a range of factors such as food and water availability and predation risk [176,182]. Bands tend to be larger in more open environments especially when resources are abundant [184]. Severe weather conditions in winter and food limitation during dry seasons causes bands to split into smaller groups [184] where they then roam in much larger and overlapping areas [185]. Adult sex ratio was important in one study showing a decrease in band size as the adult male proportion increased [186]. However, in an Argentinian study, changes in population density, rainfall and adult sex ratio did not influence group size [187]. Endoparasite burden appeared to influence group size in Shackleford Banks Island, USA [149], but not in other studies [152]. 

Within a population, bands may form a sub-population known as a herd, with similar movement patterns, a common home range, and interband dominance hierarchies that affect access to scarce resources [101]. Bands often aggregate into these larger herds around water sources, or preferred grazing sites [184]. Herd formation may reduce predation risk [182], afford shelter against harsh climatic conditions and help avoid bachelor harassment of females [178]. 

#### 5.1.3. Band Composition 

In unmanaged wild horse populations, the overall sex ratio is expected to be 1:1, with surplus males forming bachelor groups. These typically comprise males younger than 5 years, although older males can also join bachelor groups, occasionally they will be solitary [97,176,179,183]. The mean size of bachelor groups is 3, with a range of 2–17, but these are generally not stable groups [95,97,183]. 

Band composition has been shown to be stable all year-round in many different population studies across the world [97,177,179,183], with several longer-term studies of > 3 years, showing ongoing stability for many years [36,95,97,179,187,188,189,190], and less than one-third of bands dispersing within study periods [95,187,190,191]. The percentage of adult mares changing bands annually has varied from 2–30% [36,95,97,187,188,189,190,192]. Band stallion tenure may be one indicator of band stability. Mean stallion tenure is reported to be 2.8–4.4 years [95,187,190], with as long as 18 years reported [189], albeit in a managed population with a strong female-biased sex ratio. 

#### 5.1.4. Factors Influencing Social Stability 

Social stability may be influenced by water and forage availability [192], predation and adult male harassment of mares [193]. Where there is less competition for food, horses tend to live in larger relatively stable family groups, whereas those living in more arid environments where food is sparse are more often found to be in smaller unstable groups [165]. This is also observable among other equid species; when food biomass drops below 40g/m^2^ normally stable groups of plains zebras become unstable [194], and the scattered food supply in dry environments such as Ethiopian desert does not allow females to forage close enough to each other to form stable groups [20]. Dominance hierarchies exist both within and between bands. Larger groups generally obtain priority access to resources [195], although groups already in possession of the resource may have an advantage [196]. 

#### 5.1.5. Band Stallions

Stallions obtain mares in a variety of ways; acquiring unguarded mares that have dispersed or separated from their band or whose stallion has died [94,179], defeating another band stallion to take over the entire band, abducting part of a band [184], attaching themselves as a satellite to a band and eventually departing with all or some of the mares [94,184,188,197,198], or inheriting the band following death of the band stallion [180,184,199]. In most studies stallions are around 5–6 years old before they obtain their first mares [95,177,198]. 

Multi-stallion bands are also commonly reported [180], being more likely to form with lower ranking stallions of low-ranking mothers [189]. The most dominant stallion in the band is involved in most of the breeding [95,192,200]. Multi-stallion bands provide added protection, and an increased potential number of mares that the band can hold [181,192]. When faced with potential danger or rival stallions, the dominant stallion usually leads the mares or drives them away from danger, whilst the subordinate stallions place themselves between the females and the danger and confront approaching rivals [95,181,189,192,200]. Multi-stallion bands may be more stable, and mares in stable bands produce more foals than those in unstable bands [95,201]. Natality also appears to be less affected by severe winters in multi-stallion bands [202], and larger bands [197], and one study showed higher foal survival in multi-stallion bands [189]. Other studies have found no difference between single and multi-stallion bands and the number of adult mares within the band [180,200]. A New Zealand study showed mares from single-stallion bands were in better body condition than mares in multi-stallion bands, and they had higher foaling rates and lower foal mortality rates [193,200]. 

### 5.2. Population Dynamics 

#### 5.2.1. Impact of Food Resources

Food is one of the most important factors limiting wild horse population densities with many populations exhibiting density dependence and being limited by their own impact on food resources [70,90,187,203,204,205]. As forage becomes scarce, the body condition scores of horses reduce, and disease and parasitism may increase, with subsequent reductions in fecundity and survival, thereby limiting population growth [206]. 

#### 5.2.2. Birth and Survival Rates

Primiparous births typically occur from females aged 2–5 years with peak birth rates occurring for mares ages 6–17 years. When forage is abundant, mares are more likely to give birth at an earlier age [95,207], whereas when food and water become scarce, age of primiparous birth can increase and birth rates for all females can decline quickly [194]. In a range of studies across the world, the mean adult birth rate was 0.56 ± 0.001 (0.23–0.92), mean foal survival rate was 0.84 ± 0.002 (0.09–0.97), mean adult survival rate was 0.9 ± 0.001 (0.79–0.98) and mean population growth rate was 1.18 ± 0.001 (0.84–1.39) (Reviewed by [208]). Environmental conditions during early pregnancy are regarded as the most influential factor in determining whether a pregnancy will reach term [209]. Oestrous and the timing and rates of births have been correlated with rainfall in populations of free-roaming horses synchronising births with forage availability [14,187,210,211]. This may increase survival of lactating mares and their foals [49,194]. Each centimetre of rain in spring increased foal survival by 1.5% in one study, and significantly increased survival rates of horses > 20 years old but did not impact survival of adult horses up to 19 years old [212]. 

#### 5.2.3. Population Growth 

Populations can increase by up to 20% per year when resources are unlimited [213,214], but they may frequently be much lower. In free-roaming horses in New Zealand, a population growth rate of 9.6% per year was identified, and in three different wild horse populations in the Australian Alps only one of these populations was found to be increasing whilst the other two were stable, with food scarcity being the major limiting factor in population growth [70]. In France, demographics and density dependence were investigated over an eight-year period and identified resource limited population growth and decreased survival with increasing density [87]. Similarly, a seven-year study in Argentina identified a density dependent response with population growth limited by low fecundity when resources were limited [187].

#### 5.2.4. Adult Mortality 

Most wild horse populations studied exhibit relatively high survival rates with similar age structures across populations with foals comprising 8–15% of a population, juveniles 13–28% and adults 71–78% [14,207,208]. Free-roaming horses in the US have been known to live as long as 29 years [212], but longevity is extremely variable. On Assateague Island the mean age at death for stallions was 10.3 ± 0.84 years, and for mares 6.4 ± 0.85 years. However, when mares were treated with immunocontraception their longevity increased significantly with mean age at death rising to 10.2 ± 0.56 years after 1–2 years treatment and 19.9 ± 1.66 years after >3 years treatment [215]. This is presumably due to improvement in their nutrition when they are not pregnant or lactating.

Causes of adult mortality have been studied in Przewalski horses in Mongolia, with the most common causes of death being starvation (31%) and predation (22%), with other causes including injury and parasitic disease [216]. In managed free-roaming horses in a large wild horse sanctuary in Poland, reported causes of adult mortality were: drowning in marshlands where horses entered to graze the green shoots of reeds; intestinal perforation associated with ascarids; dystocia; suspected wolf predation; fractured shoulder; fractured limb; and internal haemorrhage due to a kick injury [121]. 

#### 5.2.5. Foal Mortality 

Infanticide has been reported to be the cause of 9% of foal deaths in Przewalski horses [216]. Maternal responses to infanticide risk have been studied through comparison of maternal protectiveness of foals between single and multi-stallion bands, showing that mares were more vigilant and spent more time close to foals in multi-stallion bands [85]. Much of the difference between survival rates in different populations across different regions have been attributed to local differences in predation. Large bands sizes in open environments may reduce risk of predation [217]. Many predators have been documented killing both young and adult equids throughout the world [218]. In Australia faecal analysis shows that dingoes (*Canis lupus dingo*) sometimes consume horse parts [219], but it is unclear whether this is through hunting or scavenging. There is some evidence that dingoes kill foals [110]. However, there are no scientific reports evaluating the prevalence and extent of such predation in different populations in Australia. In one study, playbacks of dingo calls did not alter maternal protectiveness suggesting that mares did not perceive dingo calls to be an immediate threat to foals [85], and foal survival has been shown to be high in studied populations in Australia [70]. In managed free-roaming horses in Poland, congenital defects were the most common cause of foal deaths, with gastric rupture and car accident also reported [121]. 

#### 5.2.6. Other Factors Influencing Population Growth Rates 

Other factors also influence population growth rates including dispersal and recruitment, and catastrophic stochastic events. Reduction in fecundity may also arise from social stress [97,207]. Due to their longevity, high survival rates, low recruitment rates, and low susceptibility to moderate environmental stressors, most wild horse populations can persist near their carrying capacity, barring catastrophic mortality events [218]. When mass mortality events do occur, populations can be relatively slow to recover because of low recruitment rates, however, sudden reductions in populations also result in a compensatory increase in reproductive rate [218,220]. Catastrophic climate events can have the greatest effect on wild horse populations with population declines of 12–61% reported after extreme cold winters in some parts of the US [113,114,116], mass mortalities during drought, with a 51% reduction reported during drought in an Australian population [14], 28% of a population dying during a thunderstorm in Argentina [115] and 4% dying with a lightning strike in Pryor Mountain, US [218]. 

### 5.3. Reproductive Physiology and Behaviours

Knowledge of reproductive physiology is particularly important for understanding impacts of nutritional, environmental and health factors on reproduction. The effects of body condition on equine reproductive physiology were recently reviewed in detail [132]. This is also important knowledge when seeking to manipulate physiology to reduce reproduction [221]. 

Mares are seasonally polyoestrous, with oestrous determined predominantly by increased day length. Additionally, nutrition, physiological condition and environmental temperature can affect seasonal reproductive activity, with conception rates impacted by age, parity, hormone metabolism, disease, trauma and genetics [132]. 

Females usually reach sexual maturity between 12 to 24 months. During the breeding season they typically complete about 12 oestrous cycles each lasting 21 days, with sexual receptiveness (oestrous) lasting 4–5 days [222]. When mares are in oestrous, they adopt a posture whereby the hind legs are abducted and tail held raised and to the side of the perineum, serving as a visual signal to attract males [223,224]. Mares exhibit an increased frequency of urination and ‘clitoral winking’, incorporating labial secretions into the urine as part of olfactory signalling [225]. Mares also exhibit a distinctive facial expression with ears back, lips pulled back, and head and neck stretched low [176]. Only sexually receptive mares will stand for mounting, whilst unreceptive mares may kick the stallion on attempts to mount [176]. 

Following a gestation period of 327–367 days [14,218,222,226], mares normally foal over spring and summer with a single foal. Mature females are capable of foaling every year, but most commonly they raise one foal every two years [222]. 

**Figure 11:**
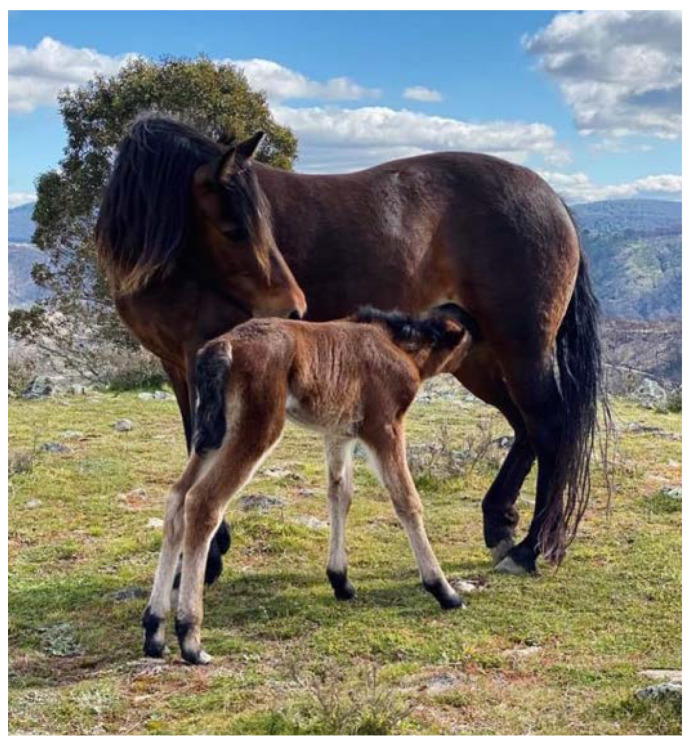
A mare nursing her newborn foal within an hour of parturition. Image A. M. Harvey.

The first successful suckling of the foal should occur within 2 h of parturition [176] (Figure 11). Suckling bout duration and frequency are reflective of the amount of milk transfer [227]. Suckling frequency decreases with increasing age of the foal. The main factor affecting lactation length is subsequent pregnancy with non-pregnant mares nursing their foals for longer than pregnant mares [228], and primiparous mares lactating longer than multiparous mares [228,229]. In one study, age of weaning did not appear to be affected by the condition of the mare [229], or number of stallions in the group [230].

### 5.4. Usual Range of Behaviours and Time Budgets 

Time budgets of wild horses have been well researched. Based on several studies in Australia, France, Japan and the US, the mean and standard error of the proportions of predominant behaviours are as follows; grazing (62.9 ± 4.79), stand resting (19.6 ± 2.76), standing (16.4 ± 1.77), recumbent (5 ± 1.95) and moving (9.5 ± 2.45) (reviewed by [165]). The daily rhythm of behaviours is linked to sunrise and sunset [231], ambient temperature [232], season [233,234], differences in forage availability and quantity [235], and the presence of insect pests [236]. Most grazing and social behaviours occur when horses are most active during the crepuscular period. Horses continue to be active during the night, with one study showing no difference in their behaviours during day and night [14], but with others reporting more resting and less feeding behaviour at night [234]. 

Typically, grazing occurs predominantly in two pulses of early morning and late afternoon, and it continues during the night. Longer periods of time are spent feeding during winter than summer [14,231,237,238]. Mares in poor body condition spend more time grazing than mares in good condition [239], and lactating mares spend more time feeding than non-lactating mares [233,240,241]. Most resting occurs during the middle of the day; recumbent rest is most common between 2 am and dawn [103,238,240]. Movement makes up a relatively small amount of the time budget, with movement time related to the distance between prime feeding areas and water sources. Stallions spend more time moving and less time feeding than mares [224,238,240]. 

During the first month after birth foals stay close to their mother, being entirely dependent on her for nutrition [242,243]. They begin to forage and interact with other horses within days of birth, and by 5 months of age their time budget becomes more like that of adult horses [240], although they spend more time in recumbent rest than adults [238,240,243].

### 5.5. Social Behaviour and Communication

Good communication is a requirement for living in large social groups. Ethograms have been well defined for wild horses, characterising the range of behaviours, facial expressions and ear, tail and general body postures involved in their communication [224,244]. 

#### 5.5.1. Affiliative Behaviours

**Figure 12:**
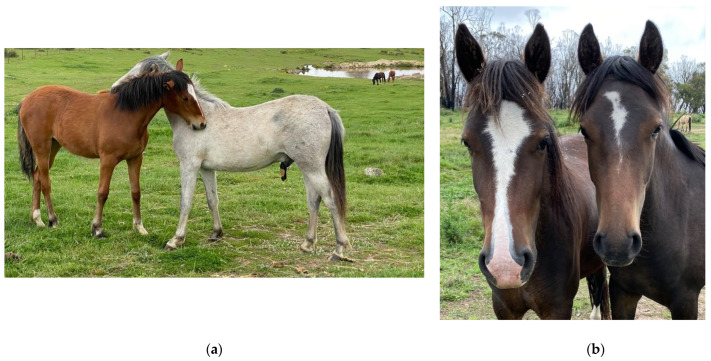
Affiliative behaviours (**a**) Allogrooming between two older foals, (**b**) Close spatial proximity between a mare and her 2-year-old filly. Images A. M. Harvey.

Affiliative behaviours include allogrooming [238,245,246] in very close proximity to each other [247] (Figure 12), or putting their heads on another’s body whilst resting [224,238]. Mutual grooming enhances social bonds, which aids stability of the band, consequently enhancing reproductive success [193,248]; it may also increase the effectiveness of protecting foals from predators. Allogrooming may also reduce heart rate suggestive of a more relaxed state [245]. Individual horses usually have preferred close companions, quantified by the proportion of time that those horses are their ‘nearest neighbour’ [247,249]; and companions usually mutually groom each other [250,251]. The nearest neighbour of foals is their mother until they are about two years old [240,251,252], and outside the foal-dam relationship, they tend to be closer to those of similar age and rank [249,250,253]. 

#### 5.5.2. Play Behaviour

Play behaviour is well described, occurring most commonly in young horses and bachelor stallions and least in adult mares [224,250,254]. Play often occurs between individuals that engage in mutual grooming [250,251], which evidently helps to reinforce social relationships [255]. Play is energetically costly, which may be why it is rarely observed in equids in arid environments [256]. Individuals that played more had better survival and body condition as yearlings despite being weaned earlier [77]. 

#### 5.5.3. Agonistic Behaviours

Agonistic behaviours such as chasing, bites, kicks and ears pinned back [224,257], being defensive or aggressive, result in an increased distance between the individuals involved [255]. Rates of agonistic behaviour tend to be low, and are affected by available space and band size, where populations that have larger available areas and larger bands, exhibit lower rates of agonistic behaviour [239]. Competition for resources is apparently the most common reason for agonistic behaviour between mares [258,259], with dominance hierarchies observed [97]. 

Older and larger horses in the band are usually more dominant [238,253,258,259,260,261]. The band stallion is usually dominant over mares, exhibited through herding behaviour [10,239], whereas dominance among mares and juveniles is most expressed through agonistic behaviours [250]. Higher rank individuals are reported to have greater reproductive success [262,263]. Despite typically being more dominant, stallions do not necessarily lead the band. This role can alternate between different mares, usually an individual of higher social rank or with increased resource needs (e.g., pregnant or lactating mare), but any band member may initiate movement of the band [264]. 

Serious aggression is rare amongst mares but can be seen between stallions and although severe injury is reportedly rare, fatal injuries can occur [265]. In one study of Przewalski horses, 18.8% of mortalities in males were attributed to injuries sustained in fights [216]. However, incursions into a band by other stallions are usually prevented by band stallions signalling their relative fighting ability [236,266]. Stable relationships between stallions and mares, and band stability can lead to increased reproductive success and lower levels of agonistic behaviour [193,230,248].

#### 5.5.4. Visual, Auditory and Olfactory Communication 

Horses communicate through visual, auditory and olfactory systems [267,268,269], with recognition of individuals enabling expression of social behaviour to conspecifics, encouraging social stability [97]. The sensory abilities of horses in relation to vision, hearing, olfaction, taste and tactile perception has recently been reviewed in detail [269]. 

Horses have a visual acuity of 20–23 cycles/degree [270], with a 60–70° anterior binocular field of view, with up to a 215° field of view out of each eye [166], using stereopsis to determine depth [270]. They are dichromate, being able to see colour especially blue and red, but not always being able to distinguish between some colours [270]. 

Vocal communications include neighing, nickering, snorting and squealing. Neighing is a contact call between horses, occurring when the horse hears another horse, usually from its social group, or when it is separated from its group [271]. Horses may process sounds from familiar individuals differently to unfamiliar sounds [272], being able to distinguish between neighs from different individuals [267]. Horses hear higher frequency (55 Hz–34 kHz) sounds than humans can, but are poor at sound localisation, only detecting differences more than 20° [270]. Nickering is a low decibel, low frequency call to encourage close contact, usually being used by dams to attract their foals. Snorting is typically due to frustration, fear, or to clear the nostrils [246], although a recent study has suggested that snorting may also be an indicator of positive emotions [273]. Squealing occurs during agonistic encounters [246]. 

Smell is also crucial for communication, with the ability to move large volumes of air through their nose in one breath, trapping large numbers of molecules containing scent information [274]. Stallions sniff mare’s urine, contact with which draws pheromones as well as volatile odours into the vomeronasal organ, communicating with brain centres coordinating the sexual response [225,246]. Curling up the upper lip, known as the flehmen response, assists these odours to make contact with the vomeronasal organ [275]. Stallions urine mark and faecal mark the faeces of other males, and on mare excretions, particularly during the breeding season [236]. Horses can differentiate between conspecifics based on their urine odour [276], and differentiate their own faeces from others, as well as recognise the relative dominance of the source of the faeces [268]. 

## 6. Concluding Comments

This is the first review to combine relevant information about horses, with a focus on free-roaming wild horses, aligned with each of the four physical/functional Domains of the Five Domains Model for assessing welfare. It therefore serves as a useful, holistic, multidisciplinary summary and point of reference for anyone wishing to assess equine welfare. Importantly, the information on free-roaming horses, obtained under optimal conditions, may be utilised to provide a better understanding of the impacts of human interventions on the welfare of horses in domestic situations. It further provides a guide for researchers of the welfare of other species, regarding how to collate this information and the breadth and depth of information required. This knowledge of species in a free-roaming state, also obtained under optimal conditions, is critical for understanding the welfare impacts of keeping these species in captivity, such as in zoos, private homes or sanctuaries.

Any changes in optimal food and water availability, environmental extremes outside of the horse’s thermoneutral zone, health, usual behaviours, and social organisation under optimal conditions may have negative impacts on welfare. Features in each of the four physical/functional Domains can serve as indicators of positive or negative welfare states [4]. The detailed information summarised in this review enables cautious inferences to be made about affective/mental experiences (in Domain 5). Domain 5 is not included in this review but will be addressed directly in subsequent publications that are in preparation, with preliminary information already available [277]. 

This novel way of synthesising the existing literature through the lens of animal welfare may also offer new insights and challenge some aspects of current wild horse ecological knowledge. Problematically, since wild horse welfare has not been scientifically assessed to date, aspects of social organisation, habitat use, behaviour and population dynamics previously reported may not always have been representative of those animals under *optimal* conditions. Welfare assessments may be misleading if data underpinning those assessments include information collected on the species in suboptimal conditions. Since the welfare of wild horses has never been scientifically evaluated to date, it is possible that there may be irregularities in what is considered to be ‘usual’. This may also have implications regarding the assessment of horse welfare in domestic situations. The authors and their colleagues have further publications on this topic in progress, derived from subsequent detailed studies of the welfare of wild free-roaming horses [277]. 

## Figures and Tables

**Table 1:** The Ten-Stage Protocol (from Harvey et al., 2020 [4]).

Acquire an understanding of the principles of Conservation Welfare. Acquire an understanding of how the Five Domains Model is used to assess welfare status. Acquire species-specific knowledge relevant to each Domain of the Model. Develop a comprehensive list of potential measurable/observable indicators in each physical domain, distinguishing between welfare status and welfare alerting indices. Select a method or methods to reliably identify individual animals. Select methods for measuring/observing the potential welfare indices and evaluate which indices can be practically measured/observed in the specific context of the study. Apply the process of scientific validation for those indices that are able to be measured/observed, and insert validated welfare status indices into the Five Domains model. Using the adjusted version of the Model that includes only the validated and practically measurable/observable welfare status indices, apply the Five Domains grading system for grading welfare compromise and enhancement within each Domain. Assign a confidence score to reflect the degree of certainty about the data on which welfare status has been graded. Including only the practically measurable/observable welfare alerting indices, apply the suggested system for grading future welfare risk within each Domain.

**Table 2:** Illustration of the species-specific information required to assess welfare of free-roaming horses using the Five Domains Model (from Harvey et al., 2020 [4]).

Domain	Species-Specific Information Required
1: Nutrition	Water requirements: volume, frequency, preferred water sources, factors influencing water requirements, adaptations to and impacts of water restriction
	Nutritional requirements and preferences
	Common nutritional deficiencies and excesses and their causes, plant toxicities
	Assessing body condition, body condition scoring systems, optimal body condition score, factors affecting body condition
2: Physical environment	Habitat preferences, and factors affecting habitat selection and use
	Preferred underfoot substrate and terrain
	Thermoneutral zone, impacts of extreme climate events, signs of thermal discomfort
3: Health	Common non-infectious diseases and their clinical signs, risk factors, aetiologies, diagnosis and prognosis
	Common infectious diseases and their clinical signs, epidemiology, mode of infection, characteristics of infectious agent (e.g., life cycle, survival in environment, involvement of other species)
	Common injuries and their clinical signs, risk factors, aetiologies, diagnosis and prognosis
	Sickness and pain behaviours
4: Behavioural interactions	Social organisation and factors affecting it
	Population dynamics
	Reproductive physiology and behaviours; oestrous, courtship, mating, gestation, parturition, lactation, maternal and newborn behaviour
	Normal range of behaviours and time budgets
	Social behaviour (including ‘rewarding behaviours’, e.g., play) and communication

**Table 3:** The Henneke 9-point body condition scoring scale (adapted from Henneke et al., 1983 [65]). Images A. M. Harvey.

Body Condition Score	Description	Example Photo
1 (Poor)	Extremely emaciated. Spinous processes, ribs, tailhead, tuber coxae and ischii projecting prominently. Bone structure of withers, shoulders and neck easily visible. No fatty tissue.	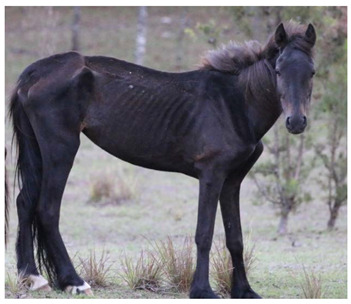
2 (Very thin)	Emaciated. Slight tissue covering over base of spinous processes. Spinous processes, ribs, tailhead, tuber coxae and ischii prominent. Withers, shoulders and neck structures visible.	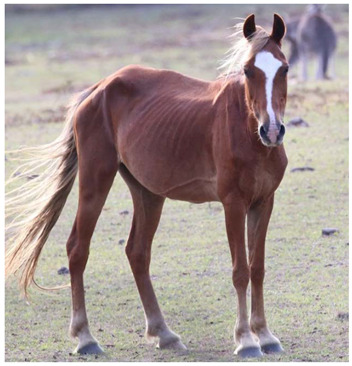
3 (Thin)	Slight fat coverage of spinous processes and ribs but still visible. Tailhead prominent, but individual vertebrae cannot be visually identified. Tuber coxae rounded but easily discernible. Withers, shoulders and neck structures visible but with some tissue coverage.	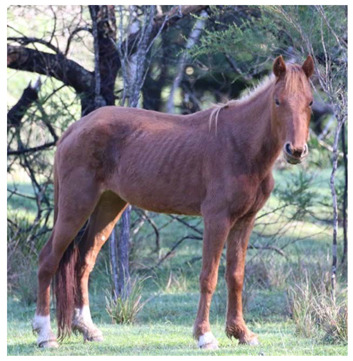
4 (Moderately thin)	Ridge of spine and outline of ribs just visible, tailhead not prominent. Tuber coxae not discernible. Withers, shoulders and neck not obviously thin.	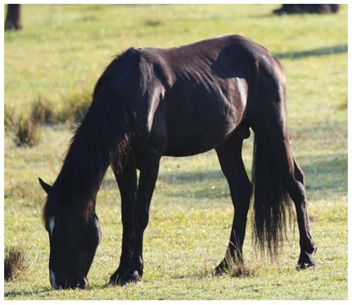
5 (Moderate)	Spine and ribs cannot be seen but ribs can be felt. Fat around tailhead. Withers rounded over spinous processes. Shoulders and neck rounded and blend smoothly into body.	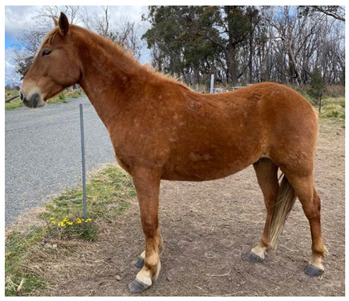
6 (Moderately fleshy)	Slight crease down spine. Fat over ribs and tailhead. Fat deposits along sides of withers, behind shoulders and along the sides of the neck.	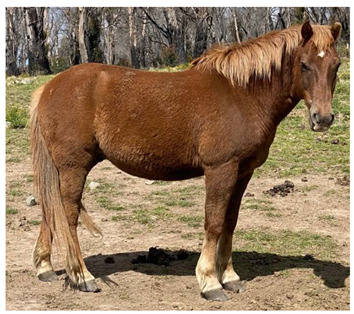
7 (Fleshy)	Crease down spine. Fat filling between ribs and around tailhead. Fat deposited along withers, behind shoulders and along the neck.	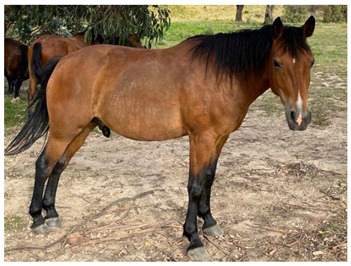
8 (Fat)	Crease down spine. Difficult to feel ribs. Fat around tailhead. Area along withers filled with fat. Fat deposits along withers and behind shoulders. Noticeable thickening of neck. Fat deposited along inner thighs.	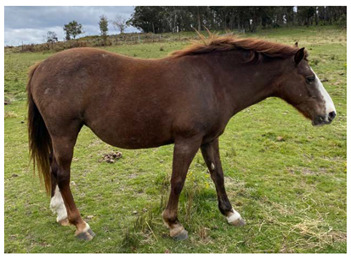
9 (Extremely fat)	Obvious crease down spine. Patchy fat appearing over ribs. Bulging fat around tailhead, along withers, behind shoulders and along neck. Fat fills in flank and is distributed along inner thighs.	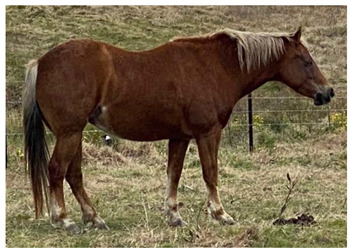

## Data Availability

Not applicable.
